# Supplementary material for: Mining LDA topics on construction engineering change risks based on graded evidence
Source: PLoS One. 2024 Jun 20;19(6):e0303424. doi: 10.1371/journal.pone.0303424 (PMC11189256; doi:10.1371/journal.pone.0303424)
Supplement: Attachment — Submitted filename: Response to Reviewers.docx [file pone.0303424.s001.docx]

Response to Reviewers

Dear editor and reviewers,

We gratefully appreciate the editors and all reviewers for their time spend making positive and constructive comments. These comments are all valuable and helpful for revising and improving our manuscript entitled "**Mining LDA topics for graded evidence on construction engineering change risks: a Chinese data analysis** (**Manuscript Number: PONE-D-23-35817**, as well as the important guiding significance to our research.

We have studied comments carefully and have made correction which we hope meet with approval. Revised portions are marked in red in the revised manuscript. The summary of corrections and the responses to the reviewer's comments are listed in the **Revision Report**.

Thank you and best regards.

Yours sincerely

Linaghai Jin

Corresponding author

Email: jinlianghai@ctgu.edu.cn

**Revision Report**

First of all, I would like to express our sincere gratitude to the reviewers for their comments. These comments are all valuable and helpful for revising and improving our manuscript, as well as the important guiding significance to our researches. We have studied comments carefully and have made correction which we hope to meet with approval. Revised portions are marked in red in the revised version. The summary of corrections and the responses to the reviewer's comments are listed below.

**Summary of the revision:**

- **Section 0&1:** We have explicitly stated the problem question and research objectives in the introduction and improved the abstract for conciseness and included a discussion of datasets and results.
- **Section 2 (Related works):** We have added a new section, Section 2: Related Works.
- **Section 3 (Method):** We have Provided a flowchart to illustrate the methodology (Fig1). In addition, we have incorporated additional citations in the methodology section to enhance the referencing of our research. Furthermore, we have provided detailed explanations regarding the data sources (Table2), parameter settings (Table3), formula interpretations, and the modeling process.
- **Section 4 (Model Results):** We explained what Gibbs sampling is and the rationale behind choosing Gibbs sampling in our research.
- **Section 5 (Evidence fusion and discussion):** We revised the managerial recommendations and added more interesting and promising discussions by the comments and suggestions from reviewers.
- **Section 6 (Conclusion):** We supplemented information about the timing of the questionnaire survey and other details and added a discussion section.

In addition, the manuscript has been carefully revised considering the language and grammar problems, including the sentences that are convoluted and hard to follow.

**Responses to reviewers**

**(**Original comments by reviewers are *in blue color***)**

***Reviewer # 1:***

***1. Comment:*** *Graded evidence? What's this?*

***1. Reply:*** Thank you for your question. Graded evidence refers to the application of the theory of graded evidence to grade the reliability of evidence in the area of engineering changes risk. We appreciate your valuable advice once again and will strive to improve the paper.

***2. Comment:*** *Remove Chinese from the title.*

***2. Reply:*** Thank you for your advice. We have revised the title to accurately and engagingly reflect it contend. We appreciate your valuable advice once again and will strive to improve the paper.

***For example:***

***original title:*** Mining LDA topics for graded evidence on construction engineering change risks: a Chinese data analysis

***revised title:*** Mining LDA topics for graded evidence on construction engineering change risks: a data analysis

***3. Comment:*** *Too many abbreviations, please use full name except for common name like LDA.*

***3. Reply:*** Thank you for your advice. We have carefully considered your suggestions regarding the use of abbreviations. Abbreviations with a frequency of less than 3 in the text have been removed to enhance the readability and clarity of the paper. This decision was made to better meet the expectations of our readers and to make our research more accessible and understandable. We appreciate your valuable advice once again and will strive to improve the paper.

***For example: (Line48)***

***Original word：***

evidence-based management (EBM)

***Revised word:***

evidence-based management

***4. Comment:*** *Something like probability of risk occurrence P and the risk importance C and calculate the average product PC of the two to determine the key risks are not needed.*

***4. Reply:*** Thank you for your advice. We've revised the abstract and removed this passage from the text. Your guidance has been instrumental, and we appreciate your thorough review.

***Original sentence：***

In addition, the questionnaire method was used to determine the probability of risk occurrence and the risk importance C and calculate the average product PC of the two to determine the key risks.

***Revised sentence:***

In addition, by combining EC risk topics with relevant literature, we identified factors influencing EC risks. Subsequently, we designed an expert survey questionnaire to determine the key risks and important risk topics associated with potential risks.

***5. Comment:*** *Synthesizing information from both class A and B evidence, not sure what is this.*

***5. Reply:*** Thank you for your comment. The mention of "Class A and B evidence" refers to the integration of information from highly credible sources. Class A represents evidence of the highest quality and reliability, such as information related to engineering change regulations. On the other hand, Class B represents evidence of higher quality or reliability, such as data related to engineering changes. The combination of this evidence grading and fusion contributes to ensuring a comprehensive analysis in our study, taking into account a variety of evidence types.

***6. Comment:*** *A total of five prominent risk themes were identified, namely contract, technology, funds, personnel, and other hazards. These are known factors for long?*

***6. Reply:*** Thank you for your advice. The identification of five prominent risk themes—contract, technology, funds, personnel, and other hazards—has been a deliberate and comprehensive effort in our study. While these factors are recognized as longstanding considerations, our aim is to provide a nuanced and up-to-date analysis that integrates both historical knowledge and contemporary insights. The inclusion of these well-established factors serves as a foundation for our research, allowing us to build upon existing understanding and explore potential developments or shifts in the dynamics of engineering change risks. We appreciate your valuable advice once again and will strive to improve the paper.

***7. Comment:*** *What is EC regulatory standards? Outsiders have no way to know what's this.*

***7. Reply:*** Thank you for raising this question. 'EC regulatory standards' refer to the legal regulations and industry standards associated with engineering changes. To enhance clarity for readers who may not be familiar with this term, I have provided a brief explanation in the abstract. This will aid in ensuring that all readers, including those unfamiliar with the specific terminology, can easily comprehend the background of our research.

***Original sentence：***

and the LDA topic model was used to analyze the EC regulatory standards and judgment documents to detect EC risk themes.

***Revised sentence:***

This involved analyzing regulations, industry standards, and judgment documents related to EC, ultimately identifying the themes associated with EC risks.

***8. Comment:*** *shorten 2.1 and do not use abbreviation like ecs.*

***8. Reply:*** Thank you for your advice. We've shortened 2.1 and revised the esc abbreviation. We appreciate your valuable advice once again and will strive to improve the paper.

***For example:***

***original title:*** Constructing a grading framework for risk evidence in ecs

***revised title:*** EC risk evidence grading framework

***9. Comment:*** *Classifying evidence according to its credibility is crucial for its scientific and pragmatic application in risk management, particularly in the sphere of EC. ? By what means?*

***9. Reply:*** Thank you for your valuable advice. The paper references the work of Haitao Zhang et al. [1] in construction safety management and prevention. Their research focuses on evidence grading, emphasizing the credibility of evidence. They define evidence in both medical and safety management fields as:

① the basis for determining facts

② materials used for demonstration

The study includes a comparative analysis of evidence sources in these domains.

Conclusively, evidence in construction safety management is classified into three levels based on credibility. Regulatory evidence, such as national laws and industry standards, holds the highest credibility, followed by literature-based evidence, including systematic reviews and practical experiences. Other evidence types encompass statements from safety organizations, expert opinions, recognition from authorities, and conference reports. We appreciate your advice and are committed to enhancing the paper.

1 Zhang H.; Wu W.; Li X.; Li Q. Research on construction site safety accident prevention system based on evidence-based approach. Chin. J. Saf. Sci. 2012, 22, 17–22. DOI: 10.16265/j.cnki.issn1003-3033.2012.12.006.

***10. Comment:*** *Line 96-104, missing citation.*

***10. Reply:*** Thank you for pointing out the missing citation. Thank you for pointing out the missing citation. We acknowledge this oversight and have added the appropriate citations in the revised version of the manuscript, specifically in previously line 96-104( Now line 169-177). We appreciate your advice and are committed to enhancing the paper.

***11. Comment:*** *The whole page where Figure 2 locates have missing citation.*

***11. Reply:*** Thank you for bringing the missing citation in the vicinity of Figure 2 to our attention. We have carefully addressed this concern and made the necessary modifications in the revised version of the manuscript. Your diligence in reviewing our work is greatly appreciated. We understand the importance of accurate and complete references and assure you that we are committed to upholding the highest standards in our research. Your valuable advice has contributed to the enhancement of our paper, and we look forward to any further guidance you may provide.(Line 243-346)

***12. Comment:*** *State what is LDA model etc when it firstly appeared in introduction and research method, the LDA section completely missed citation: Public Opinion Mining on Construction Health and Safety: Latent Dirichlet Allocation Approach, L Zeng,et al Buildings, 2023*

***12. Reply:*** Thank you for your advice. We have provided a more detailed explanation of the LDA model in both the introduction and the LDA model sections. Additionally, we have addressed the absence of citation in the LDA section by including the reference: "Public Opinion Mining on Construction Health and Safety: Latent Dirichlet Allocation Approach, L. Zeng et al., Buildings, 2023." We appreciate your valuable suggestions, and we will continue to strive for clarity and quality in the paper.

***13.Comment:*** *Results: where α = 0.1 and β = 0.01, what do these values mean?*

***13. Reply:*** Thank you for your advice. In LDA, each document is considered a mixture of multiple topics, and α is a parameter that controls the distribution of topics in a document. A higher α value leads to documents containing more topics, while a lower α value makes documents more inclined to include fewer topics. The typical range for α is a positive real number. LDA assumes that each topic is composed of multiple words, and β is a parameter that controls the distribution of words in a topic. A higher β value results in topics containing more words, while a lower β value makes topics more inclined to include fewer words. Similar to α, β is usually a positive real number.

These two parameters influence the distribution of topics in documents and the distribution of words in topics during the training process of LDA. Adjusting their values can impact the characteristics of the final topic model obtained. Setting α = 0.1 and β = 0.01 has been found to yield effective results in topic modeling. We appreciate your valuable advice once again and will strive to improve the paper.

***14. Comment:*** *State where has survey been used: Ranking of risks for existing and new building works, Sustainability, 2019*

***14. Reply:*** Thank you for your suggestion. We have added the location information in the manuscript. The questionnaire survey primarily covers the provinces of Guangdong, Hunan, Hubei, and Sichuan in China, representing diverse geographical and cultural backgrounds. This selection is aimed at ensuring our research has broad coverage in terms of diversity and representativeness. We have included the citation in the text: "Ranking of risks for existing and new building works, Sustainability, 2019." We appreciate your thorough review and valuable feedback, and we are committed to ensuring the integrity and accuracy of our research. If you have any further suggestions or concerns, please feel free to let us know.

***15. Comment:*** *The questionnaire survey sent the link to fill in the questionnaire to 26 experts? Why 26? What are criteria of experts?*

***15. Reply:*** Thank you for your inquiry. The selection of 26 experts was based on specific criteria, considering their expertise and experience in the field of construction and risk assessment. These experts were chosen due to their significant contributions to relevant research, professional background, and knowledge in the subject matter. We aimed to gather insights from a diverse group of experts to ensure a comprehensive and well-rounded understanding of the topic. The criteria for selecting experts included their academic qualifications, industry experience, and prior involvement in construction risk-related projects. We appreciate your attention to this matter and are open to any suggestions or further inquiries.

***16. Comment:*** *Add discussion section.*

***16. Reply:*** Thank you for your advice. We acknowledge the importance of including a discussion section in our paper. In the revised version, we have incorporated a dedicated discussion section to provide a comprehensive analysis of our findings, explore implications, and discuss the significance of our research in the broader context. This addition aims to enhance the overall depth and completeness of our paper. We appreciate your valuable input and strive to improve the quality of our work. If you have any further recommendations or inquiries, please feel free to inform us. We appreciate your valuable advice once again and will strive to improve the paper. (Line 459-486)

***17. Comment:*** *What is the model for 26 surveys?*

***17. Reply:*** Thank you for your advice. In our study, we employed the Likert five-point scale method to design the questionnaire. The structuring of these questions was based on the analysis results from the preceding text and aligned with relevant literature. The questionnaire, with a Cronbach’s coefficient of 0.958, surpassing 0.8, demonstrates outstanding reliability in the data. We appreciate your valuable advice once again and will strive to improve the paper.

***19. Comment:*** *Extend conclusion, limitation, direction of future research, academic, practical and policy contributions.*

***19. Reply:*** Thank you for your valuable suggestions. We acknowledge the need to expand sections such as conclusion, limitations, and future research directions in our paper. In the revised version, we have provided a more comprehensive conclusion, synthesized key findings and emphasizing their implications for both academia and practical applications. Additionally, we have elaborated on the limitations of our study, providing guidance for future research directions. The revised manuscript highlights the academic contributions of our work and its significance in practical applications, offering insights for decision-makers. We appreciate your valuable advice once again and will strive to improve the paper.

***Reviewer # 2:***

***1. Comment:*** *Dear authors, the research proposal is interesting, but I think the authors could improve the text and structure of the article before we consider it for publication. I would like to make a few suggestions:1) In the introduction, the authors did not explicitly state the problem question or the research objectives;*

***1. Reply:*** Thank you for your suggestion. In response to your comments, we have carefully reviewed the introduction section and made necessary revisions to explicitly state the problem and research objectives. The updated content now provides a clearer foundation for the study, ensuring that readers can better understand the purpose and focus of our research. We appreciate your attention to detail and are committed to enhancing the clarity and coherence of the paper. If you have any further suggestions or concerns, please feel free to let us know.

***2. Comment:*** *2) In the materials and methods section, the authors talk about data collection, but they don't mention when the data was collected, how it was collected and in which database. I would like these questions to be clarified.*

***2. Reply:*** *T*hank you for your comment. We appreciate your attention to detail. In the revised manuscript, we have provided additional information in the materials and methods section to address the timing, methods, and database used for data collection. Specifically, we now clarify when the data was collected, detail the methodologies employed in the collection process, and specify the database utilized. This additional information aims to enhance transparency and provide a comprehensive understanding of our data collection procedures. We appreciate your valuable advice once again and will strive to improve the paper.

***3. Comment:*** *3) The authors used LDA. This technique is excellent for large databases. The authors have described it, but they could provide examples of the use of LDA and a full description of the method's formulation. I suggest considering the following applications of LDA: https://doi.org/10.1108/JM2-10-2020-0268; https://doi.org/10.1108/DTA-12-2018-0109.*

***3. Reply:*** Thank you for your advice. We have carefully reviewed the two references you provided and made corresponding modifications to the section on LDA and the full description of the method's formulation in the manuscript. These changes aim to present a more comprehensive view of the application and formulation of the LDA method. We appreciate your interest in our research and are committed to ensuring the clarity and completeness of our paper. If you have any further suggestions or concerns, please feel free to let us know.

***4. Comment:*** *The authors could put together a table with all the parameters they used in LDA, such as the number of topics, and how they defined the number of topics.*

***4. Reply:*** Thank you for your suggestion. We acknowledge the importance of providing transparency regarding the parameters used in the LDA method. In the revised version of the manuscript, we have included a table detailing all the relevant parameters. The number of topics was determined in conjunction with perplexity and pyLDAvis software. LDA visualization results indicated that the topic analysis performed best under the determined number of topics in the text. This addition will enhance the reproducibility and understanding of our LDA analysis. We appreciate your attention to detail and value your input in improving the quality of our paper. If you have any further recommendations or questions, please feel free to inform us.

***Reviewer # 3:***

***1. Comment:*** *The abstract: Writing it more concisely and in a richer form is better. The end of the abstract should discuss the datasets and results.*

***1. Reply:*** Thank you for your suggestion. We acknowledge the importance of writing a concise yet comprehensive abstract. In the revised version, we have optimized the abstract to enhance conciseness and added a discussion on datasets and results at the end. These changes aim to provide a more succinct and informative overview of the research. We appreciate your valuable advice once again and will strive to improve the paper.

***2. Comment:*** *Keywords: could be more precise. Please recheck this section and write LDA in full format (Latent Dirichlet Allocation).*

***2. Reply:*** Thank you for your advice. We appreciate your suggestion regarding the precision of keywords. In response, we have carefully reviewed and refined this section, incorporating more precise keywords. Additionally, we have expanded "LDA" to its full format, "Latent Dirichlet Allocation," to enhance clarity. The revised keywords now include:

1. Architectural Engineering Change Risks

2. Evidence Grading Theory

3. Text Mining

4. Latent Dirichlet Allocation

5. Risk Management

Your input is valuable to us, and we are committed to ensuring the accuracy and effectiveness of our paper. If you have any further recommendations or inquiries, please feel free to let us know.

***3. Comment:*** *Introduction: Please write the main contributions of the research at the end of the Introduction. Please extract the literature review from the Introduction and write it in a separate section after the Introduction section with the name of the related works section.*

***3. Reply:*** Thank you for your advice. We have carefully considered your suggestion and made the necessary adjustments to the introduction section. The main contributions of the research are now highlighted at the end of the introduction. Additionally, we have extracted the literature review from the introduction and created a separate section titled "Related Works" to provide a more organized presentation of the background literature. Your guidance is appreciated, and we aim to enhance the clarity and structure of our paper. We appreciate your valuable advice once again and will strive to improve the paper.

***4. Comment:*** *The part of the related works must be completed and rewritten by recent articles from reputable journals.*

***4. Reply:*** Thank you for your advice. We have refined the section on related works based on your suggestions, incorporating recent articles from reputable journals for rewriting. We are committed to ensuring the high quality and academic standards of the paper. We appreciate your valuable advice once again and will strive to improve the paper.

***5.Comment:*** *Please consider an example from the real world to describe your method. On the other hand, this method can be better shown to the readers by a* *flow chart.*

***5. Reply:*** Thank you for your valuable feedback. We appreciate your suggestion to include a real-world example to illustrate our method. In response, we have incorporated a practical example to enhance the clarity of our methodology (Quote 23). Additionally, we have included a method analysis flowchart to visually depict the key steps and decision pathways, providing a clearer understanding for the readers. We appreciate your valuable advice once again and will strive to improve the paper.

Flow chart as follows:

***6.Comment:*** *Why did you use the Gibbs sampling method? Please explain it in the article*

***6. Reply:*** Thank you for your advice. The utilization of the Gibbs sampling method in our research is driven by its effectiveness and practicality, especially in the context of LDA. Gibbs’s sampling, as a Markov Chain Monte Carlo technique, simplifies the sampling process by updating one variable at a time during each step. This method has demonstrated notable success in handling large-scale textual data due to its efficiency and simplicity.

In our revised article, we provide a detailed explanation of the rationale behind selecting Gibbs sampling. This includes its compatibility with LDA and its advantages in dealing with the specific challenges posed by our research objectives. We appreciate your valuable advice once again and will strive to improve the paper.

***7.Comment:*** *Please explain more about how you evaluated your work by the formula. For example, how did you evaluate your data by this criterion? On what part of the data did you do this? And how? Please explain this more accurately and completely.*

***7. Reply:*** Thank you for your insightful comment. We recognize the importance of providing a detailed explanation of the evaluation process, particularly regarding the formula and its application to the data. In the revised manuscript, we have expanded the discussion on the evaluation methodology, elucidating how we applied the formula to assess the data. Specifically, we clarify the criteria used and the methodology employed in the evaluation process. This additional information aims to enhance transparency and provide readers with a clearer understanding of the evaluation of our work. We appreciate your attention to detail and value your input in improving the clarity and completeness of our paper. If you have any further recommendations or questions, please feel free to inform us.

***8.Comment:*** *Please also compare your work with the methods presented recently.*

***8. Reply:*** In the revised manuscript, we extensively compare our proposed method with the Bayesian network method widely employed in the field of risk management. This comparison aims to comprehensively understand the strengths and weaknesses of each approach and highlight the unique contributions of our method. We appreciate your suggestion, and if there are specific aspects you would like us to address in this comparison, please feel free to let us know. Your feedback is highly valuable in enhancing the quality of our research.

***9.Comment:*** *In the* *evaluation section, please explain wholly and accurately under what conditions, on what machines, and under what parameter values you compare the methods. Please mention it in some tables. It is necessary to reproduce the data by the readers of this section. Also, in this section, mention the complete information about what kind of datasets you used and the information of those datasets, as well as training data, test data, and all evaluation conditions.*

***9. Reply:*** Thank you for your insightful comment. We acknowledge the importance of providing comprehensive details on the evaluation conditions, machines, and parameter values, especially when comparing methods. However, in our study, we focused on a specific method without direct comparisons. We will address this in the revised manuscript by explicitly stating that our evaluation centered on the performance and outcomes of the proposed method itself. We will make sure to provide all relevant information about datasets, training data, test data, and any other pertinent details to enhance transparency and understanding. If you have any further suggestions or specific aspects you would like us to emphasize in this context, please feel free to share your thoughts. Your feedback is crucial in refining the quality and clarity of our research.

***10. Comment:*** *One of the fundamental questions is, what are the advantages and disadvantages of your method compared to the recent methods?*

***10. Reply:*** Thank you for your insightful inquiry. We appreciate the opportunity to elaborate on the advantages of our proposed method compared to recent Bayesian network approaches in risk analysis.

Our approach stands out in several key aspects:

- **Efficient Processing of Large-Scale Textual Data:** Unlike traditional Bayesian networks, our method, utilizing Latent Dirichlet Allocation (LDA) and evidence grading, excels in efficiently handling extensive textual data. This ensures a more thorough analysis of risk themes within the text.
- **Multilevel Evidence Grading:** We implement a multilevel evidence grading system, providing a nuanced categorization of evidence based on credibility levels. This enhances the accuracy and reliability of risk analysis by considering different levels of evidence.
- **Transparency and Scientific Foundation:** Our method emphasizes transparency and a robust scientific foundation, employing techniques like Gibbs sampling. This enhances the interpretability of risk themes, aiding decision-makers in formulating scientifically informed risk management strategies.
- **Comprehensive Risk Quantification:** Through survey questionnaires, we go beyond traditional risk analysis by quantifying the probability and degree of risks associated with engineering change. This enables the identification of key risks, providing a solid scientific basis for risk management.

These distinctive features collectively contribute to the comprehensiveness, efficiency, and depth of insights in our risk analysis method.

However, like any method, there are potential limitations to our approach. For instance, the success of our method relies on the quality and diversity of available evidence. Additionally, the applicability of the results we obtained may be influenced by specific characteristics of the dataset used. We comprehensively discuss these aspects in the revised manuscript to provide transparency and insights into the applicability of our method. Your feedback is crucial to us, and if there are specific aspects you would like us to elaborate on in this discussion, please feel free to inform us at your convenience.

***11.Comment:*** *There are some other works on semantic LDA which is not considered in this paper, such as:*

*1-https://doi.org/10.1007/s00180-022-01246-z*

*2-https://doi.org/10.1155/2022/7612276*

*3-https://doi.org/10.1111/exsy.12527*

*4-https://doi.org/10.1142/S0219622022500584*

*5-https://doi.org/10.1016/j.neucom.2022.10.002*

***11. Reply*:** Thank you for bringing these references to our attention. We appreciate your diligence in identifying additional works on semantic LDA. In the revised manuscript, we have included a comprehensive literature review that incorporates these references. This addition aims to provide a more thorough and up-to-date discussion on semantic LDA, enriching the content and contributing to the overall quality of our paper. We value your input and the improvement it brings to our work. If you have any further suggestions or concerns, please feel free to let us know.
