# Supplementary material for: Mining LDA topics on construction engineering change risks based on graded evidence
Source: PLoS One. 2024 Jun 20;19(6):e0303424. doi: 10.1371/journal.pone.0303424 (PMC11189256; doi:10.1371/journal.pone.0303424)
Supplement: Attachment — Submitted filename: renamed_04e18.docx [file pone.0303424.s002.docx]

**Responses to reviewers**

**(**Original comments by reviewers are *in blue color***)**

***Reviewer #1:***

***1. Comment:*** *The font size and format is strange.*

***1. Reply:*** Thank you for your feedback on the formatting of our manuscript. We have carefully revised the document according to the journal's formatting guidelines, including adjustments to the font size and style. We appreciate your guidance and hope the changes meet the journal's standards.

***2. Comment:*** *a data analysis in title looks strange.*

***2. Reply:*** Thank you for your feedback on our title. We've revised it to "Mining LDA topics on construction engineering change risks based on graded evidence" to better reflect our study's focus and methodology. We appreciate your guidance and are open to further suggestions.

***3. Comment:*** *2.1 Grading of evidence, the title needs to strange.*

***3. Reply:*** Thank you for your feedback regarding the title of section 2.1, "Grading of Evidence." It appears there was a misunderstanding or typographical error in your comment, as the term "needs to strange" is unclear. We interpreted this as a suggestion that the title may require adjustment for clarity or appropriateness. To address this concern, we have revised the section title to more accurately reflect the content and purpose of the discussion. The updated title is "Evidence Grading Theory," which directly aligns with the theoretical framework and methodologies discussed in this section. We hope this modification adequately addresses your comment and improves the manuscript's clarity.

***4. Comment:*** *What is The international GRADE Working Group?Full name of DIIS process should be used.*

***4. Reply:*** Thank you for your queries. The International GRADE Working Group stands for "Grading of Recommendations, Assessment, Development, and Evaluation." It is a collection of methodologists and health care professionals that have developed a systematic approach to grading the strength of evidence and the quality of recommendations in health care. This framework is widely recognized and adopted by health organizations worldwide for its clear, transparent methodology that aids in making informed health decisions.

Regarding the DIIS process, it stands for the sequence of Collecting Data (Data), Revealing Information (Information), Synthesizing Assessment (Intelligence), and Formulating a Solution (Solution). This methodological framework is utilized in our study to ensure a comprehensive and structured approach to decision-making, supporting the effective integration of evidence into the decision-making process.

We appreciate the opportunity to clarify these points and have made the necessary adjustments in the manuscript to reflect this information accurately.

***5. Comment:*** *State the research gaps reviewing the previous publications: Economic development and construction safety research: A bibliometrics approach, F Luo, Safety science, 2022*

***5. Reply:*** Thank you for your comment. We recognize the importance of F Luo's 2022 analysis on economic development and construction safety using a bibliometric approach, which we have duly cited in our study. Luo's research has provided us with a foundational understanding. Building upon this, our study aims to further investigate the risks of engineering changes in construction engineering, offering updated insights and a more detailed understanding.

We appreciate the opportunity to contribute to this important discussion and hope our research adds valuable perspectives to the field.

***6. Comment:*** *Avoid abbreviation like EC risk? What is that?*

***6. Reply:*** Thank you for your feedback regarding the use of the abbreviation "EC risk" in our manuscript. To adhere to the journal's guidelines on abbreviations, we introduced "EC risk" as a shorthand for "Engineering Change Risk," given its frequent occurrence in the text. However, recognizing your concern for clarity and the potential for confusion among readers unfamiliar with this term, we have revised the manuscript to either define "EC risk" more clearly at its first . We hope this addresses your concern and improves the readability of our study.

***7.Comment:*** *Include more studies that use text mining approach, Construction safety knowledge sharing on Twitter: A social network analysis Q Yao, Safety science, 2021*

***7. Reply:*** Thank you for your suggestion to include more studies utilizing text mining methods. We have incorporated the study by Q Yao (2021) to enrich our research on text mining. This study demonstrates the application of text mining in safety management. We hope these additions further validate the effectiveness of text mining techniques in analyzing unstructured data. We appreciate your guidance and are open to further suggestions.

***8.Comment:*** *Discussion should be based on past literatures.*

***8. Reply:*** Thank you for your constructive feedback. We acknowledge the importance of grounding our discussion in a comprehensive review of existing literature to ensure the credibility and relevance of our findings. In response to your comment, we have thoroughly revisited the literature and enriched our discussion by incorporating more references to past studies. This has allowed us to better contextualize our results within the broader field and highlight how our work builds upon and contributes to existing knowledge. We believe these enhancements have strengthened the discussion section and thank you for pointing out this opportunity for improvement.

***Reviewer #2:*** The authors extensively revised the original text in line with the guidelines and suggestions of the reviewers. When I analysed the changes made, I could see that the improvements had significantly enhanced the article. In this way, I believe that the text is, with the best judgement, in a position to be evaluated for publication.

Best regards

***Reply:***

We deeply appreciate your thorough review and positive feedback on the revisions we made to our manuscript. It is gratifying to know that the extensive modifications, guided by the insightful recommendations and guidelines provided by you and the other reviewers, have significantly improved the quality of our article. We are encouraged by your assessment and belief that our manuscript is now in a position to be considered for publication.

We are committed to further refining our work as needed to meet the journal's standards and to contribute valuable insights to the field. Your constructive comments have been instrumental in reaching this stage, and we are grateful for the time and effort you invested in reviewing our work.

Thank you once again for your support and encouragement. We look forward to the possibility of our research making a meaningful contribution to the literature.

Best regards

***Reviewer #3:*** In the revised version, the authors have considered the reviewers' comments and it is currently acceptable for publication in the journal.

***Reply:***

We sincerely thank you for finding the revised manuscript acceptable for publication in the journal. We greatly value the opinions of the reviewers and have made concerted efforts to modify the manuscript in accordance with your suggestions. Being recognized by you is incredibly encouraging.

We look forward to the successful publication of our research and hope it will bring value to the readers and researchers in the field. Thank you for your time, contribution, and for the affirmation of our work

Best regards
